# 7*H*-[1,2]Benzothia­zolo[3,2-*b*]quinazoline 5,5-dioxide

**DOI:** 10.1107/S1600536812040871

**Published:** 2012-10-06

**Authors:** Ponmudisettu Narayanan, Subramani Karthikeyan, Panayancheri Coleppa Srinivasan, Krishnan Sethusankar

**Affiliations:** a Department of Physics, RKM Vivekananda College (Autonomous), Chennai 600 004, India

## Abstract

In the title compound, C_14_H_10_N_2_O_2_S, the benzothia­zole and quinazoline ring systems are essentially planar with maximum deviations of 0.0127 (16) and 0.1588 (15) Å, respectively, and make a dihedral angleof 3.02 (5)°, which shows that the entire mol­ecule is almost planar. The O atoms deviate from the benzothia­zole ring system by 1.2231 (14) and −1.1989 (15) Å. The crystal packing features non–classical C—H⋯O hydrogen bonds and is further consolidated by π–π inter­actions [centroid–centroid distances = 3.7568 (8) and 3.8848 (9) Å].

## Related literature

For the uses and biological importance of benzothia­zole and quinazoline derivatives, see: Schwartz *et al.* (1992 ▶); Wolfe *et al.* (1990 ▶); Tereshima *et al.* (1995 ▶). For related structures, see: Khan *et al.* (2012 ▶); Grundt *et al.* (2010 ▶).
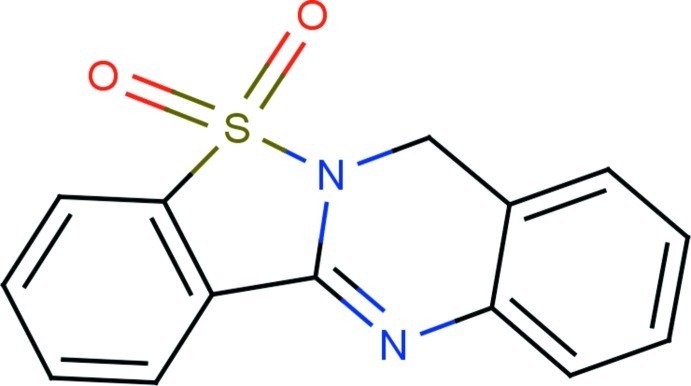


## Experimental

### 

#### Crystal data

C_14_H_10_N_2_O_2_S
*M* *_r_* = 270.31
Orthorhombic, 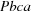

*a* = 7.9389 (2) Å
*b* = 13.1460 (4) Å
*c* = 22.7952 (7) Å
*V* = 2379.02 (12) Å^3^
*Z* = 8
Mo *K*α radiation
μ = 0.27 mm^−1^
*T* = 295 K
0.30 × 0.25 × 0.20 mm

#### Data collection

Bruker Kappa APEXII CCD diffractometer
Absorption correction: multi-scan (*SADABS*; Bruker, 2008 ▶) *T*_min_ = 0.922, *T*_max_ = 0.947
21710 measured reflections
2328 independent reflections
1981 reflections with *I* > 2σ(*I*)
*R*_int_ = 0.028

#### Refinement

*R*[*F* ^2^ > 2σ(*F* ^2^)] = 0.034
*wR*(*F* ^2^) = 0.122
*S* = 1.06
2328 reflections
172 parameters
H-atom parameters constrained
Δρ_max_ = 0.25 e Å^−3^
Δρ_min_ = −0.33 e Å^−3^

### 

Data collection: *APEX2* (Bruker, 2008 ▶); cell refinement: *SAINT* (Bruker, 2008 ▶); data reduction: *SAINT*; program(s) used to solve structure: *SHELXS97* (Sheldrick, 2008 ▶); program(s) used to refine structure: *SHELXL97* (Sheldrick, 2008 ▶); molecular graphics: *ORTEP-3* (Farrugia, 1997 ▶); software used to prepare material for publication: *SHELXL97* and *PLATON* (Spek, 2009 ▶).

## Supplementary Material

Crystal structure: contains datablock(s) global, I. DOI: 10.1107/S1600536812040871/rk2382sup1.cif

Structure factors: contains datablock(s) I. DOI: 10.1107/S1600536812040871/rk2382Isup2.hkl

Supplementary material file. DOI: 10.1107/S1600536812040871/rk2382Isup3.cml

Additional supplementary materials:  crystallographic information; 3D view; checkCIF report

## Figures and Tables

**Table 1:** Hydrogen-bond geometry (Å, °)

*D*—H⋯*A*	*D*—H	H⋯*A*	*D*⋯*A*	*D*—H⋯*A*
C2—H2⋯O1^i^	0.93	2.43	3.275 (2)	150

Symmetry code: (i) 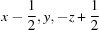.

**Fig. 1.**
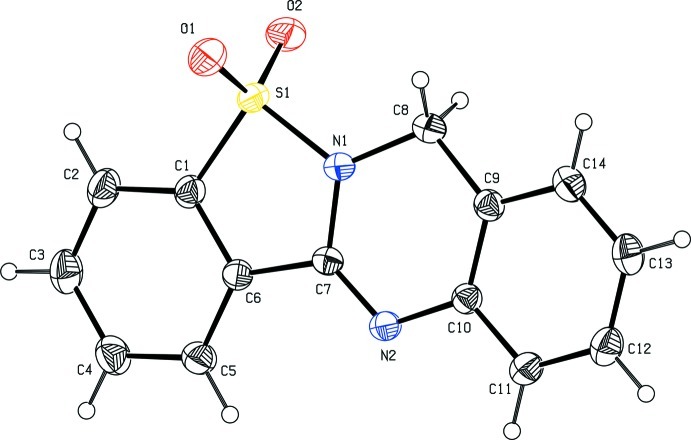
The molecular structure of the title compound with the atom numbering scheme. The displacement ellipsoids are drawn at 30% probability level. H atoms are presented as a small spheres of arbitrary radius.

**Fig. 2.**
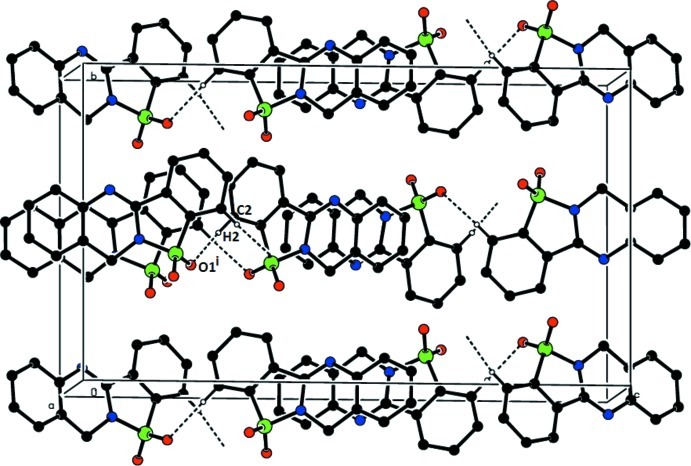
The crystal packing of the title compound, viewed down b–axis, showing C2—H2···O1i hydrogen bonds. The symmetry code: (i) -1/2+x, y, 1/2-z.

## supplementary crystallographic information

### Comment

Saccharin belongs to a class of cyclic sulfonamides and this is used as an artificial sweetener for a longtime. The benzothiazole and quinazoline derivatives form an important classes of fused heterocyclic compounds with a wide range of biological activities such as antimicrobial (Schwartz *et al.*, 1992), anticancer (Wolfe *et al.*, 1990), antiinflammatory (Tereshima *et al.*, 1995). As a part of our studies in this area, the molecular and crystal structures of the title compound have been determined and the results are presented here.

The title compound comprises a benzothiazole ring fused with quinazoline ring. X–ray analysis confirms the molecular structure and atom connectivity as illustrated in Fig. 1. The benzothiazole ring system is essentially planar with a maximum deviation of -0.0127 (16)Å for the C7 atom. The quinazoline ring system is also essentially planar with the maximum deviation of -0.1588 (15)Å for the N1 atom. The dihedral angle between the benzothiazole and quinazoline ring systems are almost coplanar with a dihedral angle of 3.02 (5)° between them.

In the benzothiazole ring system, the oxygen atoms (O1 and O2) attached to the sulfur atom are significantly deviate from the least square plane of the ring system by 1.2231 (14)Å and -1.1989 (15)Å, respectively. The thiazole ring (C1/C6/C7/N1/S1) forms a dihedral angle of 0.34 (8)° and 3.15 (6)° with the phenyl ring (C1–C6) and the quinazoline ring system, respectively. The phenyl ring (C9–C14) forms the dihedral angles of 5.01 (8)° and 5.41 (7)° with the pyrimidine ring (N1/N2/C7–C10) and the phenyl ring (C1–C6), respectively. The title compound exibits the structural similarities with already reported structures (Khan *et al.*, 2012; Grundt *et al.*, 2010).

The crystal packing is stabilized by C2—H2···O1^i^ intermolecular non–classical hydrogen bond (Table 1). The crystal packing is further stabilized by π···π interactions, with *Cg*1···*Cg*4^ii^ and *Cg*3···*Cg*4^ii^ distances being 3.7568 (8)Å and 3.8846 (9)Å, respectively, where *Cg*1 is the centre of gravity of (C1/C6/C7/N1/S1) ring; *Cg*3 is the centre of gravity of (C1–C6) ring; *Cg*4 is the centre of gravity of (C9–C14) ring. The symmetry codes: (i) -1/2+*x*, *y*, 1/2-*z*; (ii) -*x*, 1-*y*, 1-*z*. The packing view of the title compound is shown in Fig. 2.

### Experimental

A solution of Saccharin (0.91 g, 5 mmol) and *o*–amino benzyl alcohol (0.61 g, 5 mmol) in *DMF* (10 ml) was irradiated with microwaves in a 800 W domestic microwave oven for 2 minutes. The reaction solution was cooled and poured over crushed ice (200 g). The precipitated product was filtered and desiccated over anhydrous CaCl_2_. The resulting filetered product was subjected to crystallization by slow evaporation of the solvent resulting in single crystals suitable for XRD studies.

### Refinement

The positions of the hydrogen atoms were localized from the difference electron density maps and the distances were geometrically constrained. The H atoms bound to the C atoms, with d(C—H) = 0.93Å and *U*_iso_(H) = 1.2*U*_eq_(C) for aromatic, d(C—H) = 0.97Å and *U*_iso_(H) = 1.2*U*_eq_(C) for methylene.

### Crystal data

**Table:**

C_14_H_10_N_2_O_2_S	*F*(000) = 1120
*M**_r_* = 270.31	*D*_x_ = 1.509 Mg m^−^^3^
Orthorhombic, *P**b**c**a*	Mo *K*α radiation, λ = 0.71073 Å
Hall symbol: -P 2ac 2ab	Cell parameters from 2328 reflections
*a* = 7.9389 (2) Å	θ = 3.1–26.0°
*b* = 13.1460 (4) Å	µ = 0.27 mm^−^^1^
*c* = 22.7952 (7) Å	*T* = 295 K
*V* = 2379.02 (12) Å^3^	Block, yellow
*Z* = 8	0.30 × 0.25 × 0.20 mm

### Data collection

**Table:**

Bruker Kappa APEXII CCD diffractometer	2328 independent reflections
Radiation source: fine–focus sealed tube	1981 reflections with *I* > 2σ(*I*)
Graphite monochromator	*R*_int_ = 0.028
ω– & φ–scans	θ_max_ = 26.0°, θ_min_ = 3.1°
Absorption correction: multi-scan (*SADABS*; Bruker, 2008)	*h* = −9→6
*T*_min_ = 0.922, *T*_max_ = 0.947	*k* = −16→14
21710 measured reflections	*l* = −28→28

### Refinement

**Table:**

Refinement on *F*^2^	Primary atom site location: structure-invariant direct methods
Least-squares matrix: full	Secondary atom site location: difference Fourier map
*R*[*F*^2^ > 2σ(*F*^2^)] = 0.034	Hydrogen site location: inferred from neighbouring sites
*wR*(*F*^2^) = 0.122	H-atom parameters constrained
*S* = 1.06	*w* = 1/[σ^2^(*F*_o_^2^) + (0.0837*P*)^2^ + 0.4043*P*] where *P* = (*F*_o_^2^ + 2*F*_c_^2^)/3
2328 reflections	(Δ/σ)_max_ = 0.001
172 parameters	Δρ_max_ = 0.25 e Å^−^^3^
0 restraints	Δρ_min_ = −0.33 e Å^−^^3^

### Special details

**Table:**

Geometry. All s.u.'s (except the s.u. in the dihedral angle between two l.s. planes) are estimated using the full covariance matrix. The cell s.u.'s are taken into account individually in the estimation of s.u.'s in distances, angles and torsion angles; correlations between s.u.'s in cell parameters are only used when they are defined by crystal symmetry. An approximate (isotropic) treatment of cell s.u.'s is used for estimating s.u.'s involving l.s. planes.
Refinement. Refinement of *F*^2^ against ALL reflections. The weighted *R*–factor *wR* and goodness of fit *S* are based on *F*^2^, conventional *R*–factors *R* are based on *F*, with *F* set to zero for negative *F*^2^. The threshold expression of *F*^2^ > σ(*F*^2^) is used only for calculating *R*–factors(gt) *etc*. and is not relevant to the choice of reflections for refinement. *R*–factors based on *F*^2^ are statistically about twice as large as those based on *F*, and *R*–factors based on ALL data will be even larger.

### Fractional atomic coordinates and isotropic or equivalent isotropic displacement parameters (Å^2^)

**Table:**

	*x*	*y*	*z*	*U*_iso_*/*U*_eq_	
C1	0.0174 (2)	0.49618 (13)	0.32511 (7)	0.0390 (4)	
C2	−0.0613 (2)	0.51583 (15)	0.27215 (8)	0.0506 (5)	
H2	−0.0732	0.4655	0.2438	0.061*	
C3	−0.1217 (3)	0.61328 (16)	0.26314 (9)	0.0556 (5)	
H3	−0.1767	0.6288	0.2283	0.067*	
C4	−0.1014 (2)	0.68776 (16)	0.30527 (9)	0.0532 (5)	
H4	−0.1421	0.7529	0.2981	0.064*	
C5	−0.0215 (2)	0.66715 (14)	0.35795 (8)	0.0447 (4)	
H5	−0.0084	0.7177	0.3861	0.054*	
C6	0.0382 (2)	0.56991 (12)	0.36773 (7)	0.0354 (4)	
C7	0.12413 (19)	0.53087 (11)	0.42037 (7)	0.0335 (4)	
C8	0.2162 (3)	0.36384 (13)	0.46019 (8)	0.0457 (4)	
H8A	0.3027	0.3170	0.4471	0.055*	
H8B	0.1206	0.3244	0.4738	0.055*	
C9	0.2830 (2)	0.42841 (13)	0.50934 (7)	0.0365 (4)	
C10	0.24723 (19)	0.53228 (12)	0.51127 (6)	0.0342 (4)	
C11	0.3012 (2)	0.58936 (14)	0.55898 (7)	0.0433 (4)	
H11	0.2774	0.6586	0.5605	0.052*	
C12	0.3900 (2)	0.54436 (16)	0.60411 (8)	0.0497 (5)	
H12	0.4248	0.5832	0.6360	0.060*	
C13	0.4270 (2)	0.44222 (16)	0.60202 (8)	0.0498 (5)	
H13	0.4872	0.4119	0.6323	0.060*	
C14	0.3743 (2)	0.38476 (13)	0.55469 (8)	0.0449 (4)	
H14	0.4004	0.3158	0.5532	0.054*	
N1	0.1652 (2)	0.42909 (10)	0.41195 (6)	0.0414 (4)	
N2	0.15773 (18)	0.58305 (10)	0.46603 (6)	0.0371 (3)	
O1	0.24864 (17)	0.35324 (11)	0.31425 (6)	0.0575 (4)	
O2	−0.01803 (19)	0.30315 (10)	0.35705 (6)	0.0581 (4)	
S1	0.10439 (6)	0.38070 (3)	0.348166 (18)	0.04023 (19)	

### Atomic displacement parameters (Å^2^)

**Table:**

	*U*^11^	*U*^22^	*U*^33^	*U*^12^	*U*^13^	*U*^23^
C1	0.0408 (9)	0.0411 (9)	0.0352 (9)	−0.0072 (7)	0.0019 (7)	−0.0004 (6)
C2	0.0551 (11)	0.0603 (11)	0.0363 (9)	−0.0090 (10)	−0.0058 (8)	−0.0043 (8)
C3	0.0545 (12)	0.0697 (13)	0.0425 (10)	−0.0024 (10)	−0.0114 (8)	0.0090 (9)
C4	0.0570 (12)	0.0512 (11)	0.0514 (11)	0.0031 (9)	−0.0086 (8)	0.0096 (9)
C5	0.0487 (10)	0.0390 (9)	0.0464 (9)	0.0016 (8)	−0.0060 (8)	0.0006 (7)
C6	0.0359 (8)	0.0360 (8)	0.0343 (8)	−0.0053 (7)	0.0003 (6)	0.0003 (6)
C7	0.0338 (8)	0.0296 (7)	0.0373 (8)	−0.0023 (6)	0.0014 (6)	−0.0002 (6)
C8	0.0606 (11)	0.0329 (8)	0.0436 (9)	0.0040 (8)	−0.0039 (8)	0.0006 (7)
C9	0.0351 (8)	0.0373 (8)	0.0370 (8)	0.0003 (7)	0.0051 (6)	0.0029 (6)
C10	0.0322 (8)	0.0360 (8)	0.0345 (8)	−0.0005 (7)	0.0016 (6)	0.0005 (6)
C11	0.0472 (10)	0.0428 (9)	0.0401 (9)	−0.0004 (8)	−0.0019 (7)	−0.0042 (7)
C12	0.0515 (11)	0.0588 (12)	0.0387 (9)	−0.0042 (9)	−0.0051 (8)	−0.0030 (8)
C13	0.0481 (10)	0.0595 (12)	0.0418 (9)	0.0005 (9)	−0.0062 (8)	0.0107 (8)
C14	0.0456 (10)	0.0424 (10)	0.0468 (10)	0.0029 (8)	0.0014 (8)	0.0085 (7)
N1	0.0576 (9)	0.0303 (7)	0.0362 (7)	0.0016 (7)	−0.0044 (6)	−0.0028 (5)
N2	0.0417 (7)	0.0335 (7)	0.0363 (7)	0.0022 (6)	−0.0034 (6)	−0.0021 (5)
O1	0.0659 (9)	0.0564 (8)	0.0501 (8)	0.0070 (7)	0.0156 (6)	−0.0062 (6)
O2	0.0750 (10)	0.0423 (7)	0.0570 (8)	−0.0208 (7)	0.0046 (7)	−0.0050 (6)
S1	0.0516 (3)	0.0345 (3)	0.0346 (3)	−0.00603 (18)	0.00492 (17)	−0.00507 (14)

### Geometric parameters (Å, º)

**Table:**

C1—C6	1.382 (2)	C8—H8A	0.9700
C1—C2	1.384 (2)	C8—H8B	0.9700
C1—S1	1.7485 (18)	C9—C14	1.387 (2)
C2—C3	1.383 (3)	C9—C10	1.395 (2)
C2—H2	0.9300	C10—C11	1.389 (2)
C3—C4	1.381 (3)	C10—N2	1.419 (2)
C3—H3	0.9300	C11—C12	1.380 (3)
C4—C5	1.384 (2)	C11—H11	0.9300
C4—H4	0.9300	C12—C13	1.375 (3)
C5—C6	1.382 (2)	C12—H12	0.9300
C5—H5	0.9300	C13—C14	1.382 (3)
C6—C7	1.473 (2)	C13—H13	0.9300
C7—N2	1.275 (2)	C14—H14	0.9300
C7—N1	1.390 (2)	N1—S1	1.6589 (14)
C8—N1	1.452 (2)	O1—S1	1.4281 (13)
C8—C9	1.502 (2)	O2—S1	1.4230 (13)
			
C6—C1—C2	122.42 (17)	C14—C9—C8	120.43 (16)
C6—C1—S1	110.51 (12)	C10—C9—C8	120.31 (14)
C2—C1—S1	127.06 (14)	C11—C10—C9	119.36 (15)
C3—C2—C1	117.32 (18)	C11—C10—N2	118.00 (14)
C3—C2—H2	121.3	C9—C10—N2	122.63 (14)
C1—C2—H2	121.3	C12—C11—C10	120.64 (17)
C2—C3—C4	120.86 (18)	C12—C11—H11	119.7
C2—C3—H3	119.6	C10—C11—H11	119.7
C4—C3—H3	119.6	C13—C12—C11	120.12 (17)
C3—C4—C5	121.19 (18)	C13—C12—H12	119.9
C3—C4—H4	119.4	C11—C12—H12	119.9
C5—C4—H4	119.4	C12—C13—C14	119.74 (16)
C6—C5—C4	118.57 (17)	C12—C13—H13	120.1
C6—C5—H5	120.7	C14—C13—H13	120.1
C4—C5—H5	120.7	C13—C14—C9	120.93 (16)
C1—C6—C5	119.62 (15)	C13—C14—H14	119.5
C1—C6—C7	112.56 (14)	C9—C14—H14	119.5
C5—C6—C7	127.81 (15)	C7—N1—C8	121.96 (13)
N2—C7—N1	125.55 (14)	C7—N1—S1	114.95 (11)
N2—C7—C6	125.08 (14)	C8—N1—S1	121.22 (11)
N1—C7—C6	109.36 (13)	C7—N2—C10	116.43 (13)
N1—C8—C9	109.23 (14)	O2—S1—O1	116.34 (9)
N1—C8—H8A	109.8	O2—S1—N1	110.41 (8)
C9—C8—H8A	109.8	O1—S1—N1	109.76 (8)
N1—C8—H8B	109.8	O2—S1—C1	113.27 (9)
C9—C8—H8B	109.8	O1—S1—C1	111.92 (8)
H8A—C8—H8B	108.3	N1—S1—C1	92.60 (7)
C14—C9—C10	119.20 (15)		
			
C6—C1—C2—C3	−0.7 (3)	C12—C13—C14—C9	−0.6 (3)
S1—C1—C2—C3	179.80 (15)	C10—C9—C14—C13	1.3 (3)
C1—C2—C3—C4	0.9 (3)	C8—C9—C14—C13	−175.78 (16)
C2—C3—C4—C5	−0.6 (3)	N2—C7—N1—C8	15.1 (3)
C3—C4—C5—C6	0.1 (3)	C6—C7—N1—C8	−165.98 (16)
C2—C1—C6—C5	0.2 (3)	N2—C7—N1—S1	179.66 (14)
S1—C1—C6—C5	179.80 (13)	C6—C7—N1—S1	−1.41 (17)
C2—C1—C6—C7	179.50 (15)	C9—C8—N1—C7	−22.8 (2)
S1—C1—C6—C7	−0.94 (18)	C9—C8—N1—S1	173.54 (12)
C4—C5—C6—C1	0.1 (3)	N1—C7—N2—C10	1.1 (2)
C4—C5—C6—C7	−179.04 (16)	C6—C7—N2—C10	−177.62 (13)
C1—C6—C7—N2	−179.58 (16)	C11—C10—N2—C7	173.48 (15)
C5—C6—C7—N2	−0.4 (3)	C9—C10—N2—C7	−6.2 (2)
C1—C6—C7—N1	1.48 (19)	C7—N1—S1—O2	−115.25 (13)
C5—C6—C7—N1	−179.33 (16)	C8—N1—S1—O2	49.45 (17)
N1—C8—C9—C14	−165.40 (15)	C7—N1—S1—O1	115.20 (13)
N1—C8—C9—C10	17.6 (2)	C8—N1—S1—O1	−80.10 (16)
C14—C9—C10—C11	−1.1 (2)	C7—N1—S1—C1	0.78 (14)
C8—C9—C10—C11	175.98 (16)	C8—N1—S1—C1	165.48 (15)
C14—C9—C10—N2	178.63 (15)	C6—C1—S1—O2	113.68 (13)
C8—C9—C10—N2	−4.3 (2)	C2—C1—S1—O2	−66.78 (18)
C9—C10—C11—C12	0.2 (3)	C6—C1—S1—O1	−112.40 (13)
N2—C10—C11—C12	−179.50 (15)	C2—C1—S1—O1	67.14 (18)
C10—C11—C12—C13	0.5 (3)	C6—C1—S1—N1	0.13 (14)
C11—C12—C13—C14	−0.3 (3)	C2—C1—S1—N1	179.66 (17)

### Hydrogen-bond geometry (Å, º)

**Table:**

*D*—H···*A*	*D*—H	H···*A*	*D*···*A*	*D*—H···*A*
C2—H2···O1^i^	0.93	2.43	3.275 (2)	150

Symmetry code: (i) *x*−1/2, *y*, −*z*+1/2.
